# Incidence of RSV in Adults: A Comprehensive Review of Observational Studies and Critical Gaps in Information

**DOI:** 10.1093/infdis/jiae314

**Published:** 2024-06-27

**Authors:** Benjamin Doty, Parinaz Ghaswalla, Rhonda L Bohn, Sonia K Stoszek, Catherine A Panozzo

**Affiliations:** Bohn Epidemiology, LLC, Boston, Massachusetts, USA; Moderna, Inc, Cambridge, Massachusetts, USA; Bohn Epidemiology, LLC, Boston, Massachusetts, USA; Moderna, Inc, Cambridge, Massachusetts, USA; Moderna, Inc, Cambridge, Massachusetts, USA

**Keywords:** RSV, literature review, incidence, adults, gap analysis

## Abstract

**Background:**

We conducted a global comprehensive literature review of observational studies reporting respiratory syncytial virus (RSV) incidence in adults and determined current evidence gaps.

**Methods:**

PubMed and Embase were searched for English-language publications (2000–2022) and congress abstracts (2019–2021) reporting RSV incidence rates/cumulative incidence. Cross-sectional studies, case series, and other designs estimating only RSV frequency were excluded. The search included all geographic areas; data were extracted by age group and underlying condition where available.

**Results:**

In total, 528 potentially relevant records were identified, of which 37 primary studies were relevant to this review. Most evidence was from high-income regions. Approximately two-thirds of the studies reported RSV incidence in the hospital setting. Fifteen studies included or focused exclusively on RSV incidence in adult populations with underlying conditions. Studies varied in their measurement and presentation of incidence. RSV incidence estimates were highly variable within and between geographic regions. Overall, RSV incidence tended to increase with age and was highest in adults with underlying conditions.

**Conclusions:**

Estimates of RSV incidence are highly variable across populations and geographies. Further population-based studies with well-defined, consistent case definitions and surveillance strategies are needed for accurate and comparable estimates of RSV incidence, particularly in the geographic regions identified by the gap analysis.

## Teaser

We conducted a global comprehensive literature review of studies reporting RSV incidence in adults. Estimates of RSV incidence are variable; population-based studies with well-defined case definitions and surveillance strategies are needed in addition to studies in community-based populations and lower- and middle-income regions.

Respiratory syncytial virus (RSV) is a seasonal virus and a common cause of lower respiratory tract disease in infants, young children, older adults, and those with underlying conditions that increase the risk for severe RSV disease [1–4]. RSV infections occur throughout winter in temperate climates and throughout the year in tropical climates, with a pattern of outbreaks during the hot, humid, rainy summer period [5, 6]. Globally, among adults aged ≥65 years, 336 000 RSV acute respiratory infection (ARI) hospitalizations and 14 000 RSV-ARI in-hospital deaths occurred in 2015 [1]. The risk of serious outcomes following RSV infection in adults aged ≥60 years is at least double for individuals with chronic obstructive pulmonary disease (COPD) or congestive heart failure (CHF) compared with individuals without these conditions [7, 8]. Currently, 2 prefusion F-based RSV vaccines are approved in the United States and European Union for the prevention of RSV in adults aged ≥60 years [9–11].

Primary studies of RSV occurrence in adults typically report estimates by country, area, or age group [1, 12]. Reviews that include data from multiple countries, areas, and age groups are needed to guide future observational studies on RSV incidence and vaccination strategies by, for example, identifying regions and subpopulations at most need for a vaccine. Therefore, we performed a global comprehensive literature review of observational studies of RSV incidence in adults (≥18 years), including studies conducted in community and medically attended populations as well as populations with selected underlying conditions that increase the risk for severe RSV disease [2, 3, 13, 14]. Additionally, we identified evidence gaps by age group and geography to inform future research.

## METHODS

PubMed and Embase searches were conducted to identify English-language studies reporting RSV incidence in adults (1 January 2000 to 30 May 2022; Table 1), using combinations of terms for RSV and incidence as search terms. The most relevant publications identified were used to find additional records from the PubMed Similar Articles and Web of Science Cited References. Embase was searched for records from indexed conferences (2019–2021). RSV Foundation 2021 (ReSViNET) conference abstracts were also searched.

**Table 1.** RSV Incidence Rates From Studies in Community and Medically Attended Populations^a^

Citation	Region	Country	Diagnostic Method	Study Description^b^	Age, y	Incidence Estimates^c,d^
Studies in community populations						
Korsten et al (2021) [15]	Europe	Belgium Netherlands United Kingdom	PCR, surveillance	2017–2019 Prospective cohorts N = 1040 total (n = 527 in 2017–2018 cohort; n = 513 in 2018–2019 cohort)		Cumulative incidence, 2017–2018
					≥60	2.1% (1.0%–3.7%)
						Cumulative incidence, 2018–2019
					≥60	4.9% (3.2%–7.1%)
Kumar et al (2021) [16]	Asia	India	PCR, surveillance	2015–2017 Prospective cohort N = 1403 (n = 606 cases of LRTI; n = 18 cases of LRTI-associated RSV)		Incidence per 100 000 PYs
					60–64	260 (50–810)
					65–74	760 (240–1470)
					≥60	620 (230–980)
					≥75	1010 (270–2230)
Praphasiri et al (2021) [17]	Asia	Thailand	PCR, surveillance	2015–2017 Prospective cohort N = 3220 (n = 2907 participants without underlying CPC [n = 68 cases of RSV]; n = 313 participants with underlying CPC [n = 13 cases of RSV])		Incidence per 100 000 PYs
					≥65	1230 (960–1560) among participants without CPC
					≥65	2320 (1250–3950) among participants with CPC
Studies in medically attended populations						
Branche et al (2022) [8]	North America	United States	PCR, surveillance	2017–2020 Surveillance (hospital-based) N = 10 860 who met case definition for RSV testing; n = 1039 patients with RSV		Ranges of seasonal incidence per 100 000 population (hospitalized)
					18–49	7.8 (4.9–12.4) to 11.9 (7.4–19.2)
					50–64	33.5 (20.8–53.8) to 57.5 (43.8–75.5)
					65–74	83.2 (60.3–114.9) to 126.2 (83.9–189.9)
					75–84	155.0 (111.3–215.8) to 281.4 (196.9–402.3)
					≥18	44.2 (38.0–51.5) to 58.9 (49.7–69.7)
					≥65	136.9 (113.3–165.5) to 255.6 (207.4–314.9)
					≥85	207.2 (137.7–311.6) to 666.2 (483.2–918.4)
Jackson et al (2021) [24]	North America	United States	PCR, HCP	2011–2016 Surveillance (hospital-based) N = 82 000–162 000 (range) patients per cohort per year		Mean annual incidence per 100 000 population (outpatient)
					18–30	850 (220–1710)
					31–49	1090 (330–1860)
					50–64	1450 (550–2450)
					≥65	2320 (1110–3680)
McClure et al (2014) [25]	North America	United States	PCR, HCP	2006–2010 Prospective cohort N = 20 453 (n = 164 cases of RSV across the study period)		Seasonal incidence per 100 000 population (outpatient)
					50–59	1240 (990–1560)
					60–69	1470 (1100–1960)
					≥70	1990 (1530–2580)
Mesa-Frias et al (2022) [27]	North America	United States	ICD-9 or ICD-10	2000–2020 Surveillance (hospital-based) Two data sources: Optum and MarketScan		Annual range of incidence per 100 000 population based on Optum data (outpatient)
					60–64	25.2–66.1
					≥65	37.3–75.5
					≥85	92.4–140.6
						Annual range of incidence per 100 000 population based on MarketScan data (outpatient)
					60–64	31.9–82.1
					≥65	54.1–97.3
					≥85	79.2–234.7
Nolen et al (2020) [30]	North America	United States	PCR, surveillance	2016–2018 Surveillance (hospital-based)		Annual incidence per 100 000 population (hospitalized)
					18–49	30 (11–65)
					50–64	152 (73–280)
					≥65	356 (178–637)
Sieling et al (2021) [36]	North America	United States	PCR, HCP	2017–2019 Retrospective chart review 2017–2018: N = 2043 patients, of which 198 (9.2%) had RSV 2018–2019: N = 2189 patients, of which 243 (10.5%) had RSV		Incidence per 100 000 population, 2017–2018 (hospitalized)
					18–49	9
					50–64	55
					65–79	110
					≥18	49
					≥80	392
						Incidence per 100 000 population, 2018–2019 (hospitalized)
					18–49	13
					50–64	60
					65–79	145
					≥18	58
					≥80	401
Tong et al (2020) [38]	North America	United States	ICD-9-CM	2008–2014 Retrospective chart review using the Truven Health MarketScan Commercial Claims and Encounters database and the Medicare database N = 48 million individuals per year (on average); 46 000–77 000 cases of RSV were diagnosed annually		Mean annual incidence per 100 000 population (hospitalized/outpatient)
					18–49	80
					50–64	150
					65–74	330
					75–84	550
					≥85	810
Widmer et al (2012) [40]	North America	United States	PCR, HCP	2006–2009 Surveillance (hospital-based) N = 591 patients, of whom 31 had RSV		Mean annual incidence per 100 000 population (hospitalized)
					50–64	82 (33–123)
					≥50	150 (86–198)
					≥65	254 (131–380)
Widmer et al (2014) [39]	North America	United States	PCR, HCP	2009–2010 Surveillance (hospital-based) N = 1248 patients; n = 32 cases of RSV		Incidence per 100 000 population, 2009–2010 (hospitalized)
					18–49	21.1 (10–42)
					50–64	67.1 (33–134)
					≥18	55 (37–81)
					≥50	112.4 (71–177)
					≥65	189.6 (104–340)
						Incidence per 100 000 population, 2009–2010 (outpatient)
					18–49	131.8 (67–253)
					50–64	127.6 (44–354)
					≥18	154.4 (93–254)
					≥50	194.8 (90–408)
					≥65	339.6 (117–908)
Schanzer et al (2008) [34]	North America	Canada	ICD-9-CM	1994–2000 Canadian National Hospitalization Morbidity Database N = 170 000 hospitalizations/year		Average annual incidence per 100 000 population (hospitalized)
					20–49	10
					50–64	23
					≥65	108
McCracken et al (2013) [26]	North America	Guatemala	PCR, surveillance	2007–2011 Surveillance (hospital-based) N = 2565 clinic patients, of which 300 had RSV		Incidence per 100 000 PYs (hospitalized)
					18–49	3
					50–64	13
					≥50	20
					≥65+	29
						Incidence per 100 000 PYs (outpatient)
					18–49	44
					50–64	15
					≥50	13
					≥65	19
Chavez et al (2019) [20]	South America	Bolivia	PCR and/or IF, surveillance	2012–2017 Surveillance (hospital-based) N = 2592 cases of SARI, of which 931 were among adults; the number of cases of RSV among adults in the sample was not reported		Incidence per 100 000 population, 2013 (hospitalized)
					20–49	80 (10–570)
					50–64	80 (10–570)
					≥65	780 (110–5530)
						Incidence per 100 000 population, 2014 (hospitalized)
					20–49	170 (70–470)
					50–64	170 (70–470)
					≥65	610 (90–4310)
						Incidence per 100 000 population, 2015 (hospitalized)
					20–49	0
					50–64	0
					≥65	1040 (150–7380)
						Incidence per 100 000 population, 2016 (hospitalized)
					20–49	60 (10–430)
					50–64	60 (10–430)
					≥65	0
Sharp et al (2022) [35]	Europe	England	Variable: antigen, culture, or genomic (PCR/LCR), HCP	2010–2017 Surveillance (hospital-based)		Average seasonal incidence per 100 000 population (hospitalized)
					6574	71 (52–90)
					≥75	251 (186–316)
Fleming et al (2015) [22]	Europe	United Kingdom	ICD-10	1995–2009 Surveillance (hospital-based) National data obtained from Public Health England, Clinical Practice Research Datalink, Hospital Episode Statistics, and the Office of National Statistics databases		Average seasonal incidence (range) per 100 000 population (hospitalized)
					18–49	4 (3–5)
					50–64	30 (22–36)
					65–74	86 (62–101)
					≥75	234 (180–291)
						Average seasonal incidence (range) per 100 000 population (outpatient)
					18–49	677 (443–850)
					50–64	1325 (928–1542)
					65–74	1742 (1259–2038)
					≥75	2175 (1554–2516)
Subissi et al (2021) [37]	Europe	Belgium	PCR	2015–2019 Surveillance (hospital-based) N = 2105 cases with SARI in older adults, of which 100 had RSV		Incidence per 100 000 PYs (hospitalized)
					≥65	46.8 (38.4–57.6)
Rowlinson et al (2013) [32]	Africa	Egypt	PCR	2009–2012 Surveillance (hospital- and clinic-based) N = 5342 hospitalized patients, of which 1672 were adults (aged 20+ years) and 3 had RSV N = 771 outpatients, of which 56 were adults (aged 20+ years) and 8 had RSV		Incidence per 100 000 PYs (hospitalized)
					20–49	1.9 (0.2–3.0)
					50–64	6.5 (4.0–11.0)
					≥50	6.2 (0.4–10.0)
					≥65	5.0 (2.0–13.0)
						Incidence per 100 000 PYs (outpatient)
					20–49	517 (325–3075)
					50–64	NR
					≥50	0
					≥65	NR
Bigogo et al (2013) [18]	Africa	Kenya	PCR	2007–2011 Surveillance (community-based) Two sites: Lwak (approximately 27 000 participants in 2 rural villages) and Kibera (approximately 25 000 participants in 33 urban villages [“slum”])		Incidence of RSV-associated SARI per 100 000 PYs
					≥18	440 in Lwak site; 80 in Kibera site
						Incidence of RSV-associated ILI per 100 000 PYs
					≥18	0 in Lwak site; 10 in Kibera site
Emukule et al (2014) [21]	Africa	Kenya	PCR	2009–2012 Surveillance (hospital-based) N = 5507 hospitalized patients (n = 176 participants with RSV in the study [not disaggregated by age] N = 1632 outpatients (n = 101 participants with RSV in study [not disaggregated by age])		Average annual incidence per 100 000 population with SARI (hospitalized)
					18–34	10 (0–50)
					35–49	0 (0–130)
					≥50	270 (190–390)
						Average annual incidence per 100 000 population with ILI (outpatient)
					18–34	20 (0–490)
					35–49	NR
					≥50	NR
Moyes et al (2017) [28]	Africa	South Africa	PCR	2009–2013 Surveillance (hospital-based) N = 7872, of which 66 cases of RSV were identified among participants without HIV		Incidence per 100 000 population, 2010 (hospitalized)
					18–44	6 (4–8)
					45–64	9 (6–15)
					≥65	19 (10–33)
						Incidence per 100 000 population, 2011 (hospitalized)
					18–44	5 (3–7)
					45–64	12 (7–18)
					≥65	21 (11–35)
						Incidence per 100 000 population, 2012 (hospitalized)
					25–44	6 (4–9)
					45–64	16 (11–23)
					≥65	19 (11–33)
Chan et al (2015) [29]	Asia	China	IF	1998–2012 Retrospective cohort N = 4839 cases of RSV across the 15-year study period (the distribution of cases by age group was NR)		Incidence per 100 000 PYs (hospitalized)
					≥65	57
Fry et al (2010) [23]	Asia	Thailand	PCR or serologic test	2003–2007 Surveillance (hospital-based) N = 10 868, of which 105 were cases of RSV among adults		Incidence per 100 000 PYs (hospitalized)
					20–49	3.6 (2.4–4.9)
					50–64	8.6 (5.2–12)
					≥65	39 (28–50)
Naorat et al (2013) [29]	Asia	Thailand	PCR	2008–2011 Surveillance (hospital-based) N = 13 982, of which 1137 had RSV		Incidence per 100 000 PYs (hospitalized)
					20–49	9 (6–11)
					50–64	40 (32–49)
					≥65	130 (108–152)
Saravanos et al (2019) [33]	Oceania	Australia	ICD-10-AM	2011–2015 Retrospective chart review N = 2657 hospitalizations with RSV as a principal diagnosis among adults ages 25+ years (2006–2015)		Mean annual incidence per 100 000 population (indigenous) (hospitalized)
					25–34	1
					35–44	2
					45–54	4
					55–64	4
					≥65	8
						Mean annual incidence per 100 000 population (nonindigenous) (hospitalized)
					25–34	<0.5
					35–44	1
					45–54	1
					55–64	2
					≥65	9
Prasad et al (2020) [31]	Oceania	New Zealand	PCR	2012–2015 Retrospective chart review N = 731 204 adults (annual average) obtained from national administrative datasets; n = 348 RSV-associated hospitalizations		Seasonal incidence per 100 000 population (hospitalized)
					18–49	5.9 (4.3–7.5)
					50–64	24.2 (18.2–30.2)
					65–79	72.9 (57.4–88.3)
					≥18	23.6 (21.0–26.1)
					≥65	99.2 (82.4–115.9)
					≥80	190.8 (137.6–244.0)
Studies in mixed populations						
Kurai et al (2022) [42]	Asia	Japan	PCR	2019–2020 Prospective cohort N = 1000		Annual cumulative incidence
					65–74	1.40% (95% CI NR)
					75–84	0.80% (95% CI NR)
					≥65	2.40% (1.54%–3.55%)
					≥85	0.20% (95% CI NR)
Falsey et al (2005) [41]	North America	United States	PCR, culture, or serologic test	1999–2003 Prospective cohort (3 cohorts) Cohort 1: n = 608 healthy adults (ages 65+ years) who did not have an underlying disabling condition (COPD or CHF) Cohort 2: n = 1388 hospitalized adults Cohort 3: n = 540 high-risk adults (aged 21+ years) who had an underlying condition (COPD or CHF)		Incidence per 100 000 PYs
					21+	18 000
					≥65	10 800
						Annual cumulative incidence
					All	5.5%
	

Abbreviations: ARI, acute respiratory illness; CAD, coronary artery disease; CHF, congestive heart failure; CI, confidence interval; COPD, chronic obstructive pulmonary disease; CPC, cardiopulmonary condition; CRPD, Clinical Practice Research Datalink; CVD, cardiovascular disease; DM, diabetes mellitus; ED, emergency department; HCP, healthcare provider; ID, identification; IF, immunofluorescence; ILI, influenza-like illness; LCR, ligase chain reaction; LRTI, lower respiratory tract infection; MS, multiple sclerosis; N, sample size; NR, not reported; PY, person-year; RSV, respiratory syncytial virus; SARI, severe acute respiratory illness.

^a^Table is ordered by region and country, alphabetically.

^b^Study period is given as year(s) over which time the study was conducted.

^c^Incidence estimates listed are incidence rates or cumulative incidence, as available in the original publications. Unless otherwise stated, incidence estimates are given per 100 000 population or per 100 000 person-years, based on available study data.

^d^Interval estimates are reported if available from the original study source documents. Interval estimates are 95% confidence intervals unless otherwise stated.

**Figure 1.**
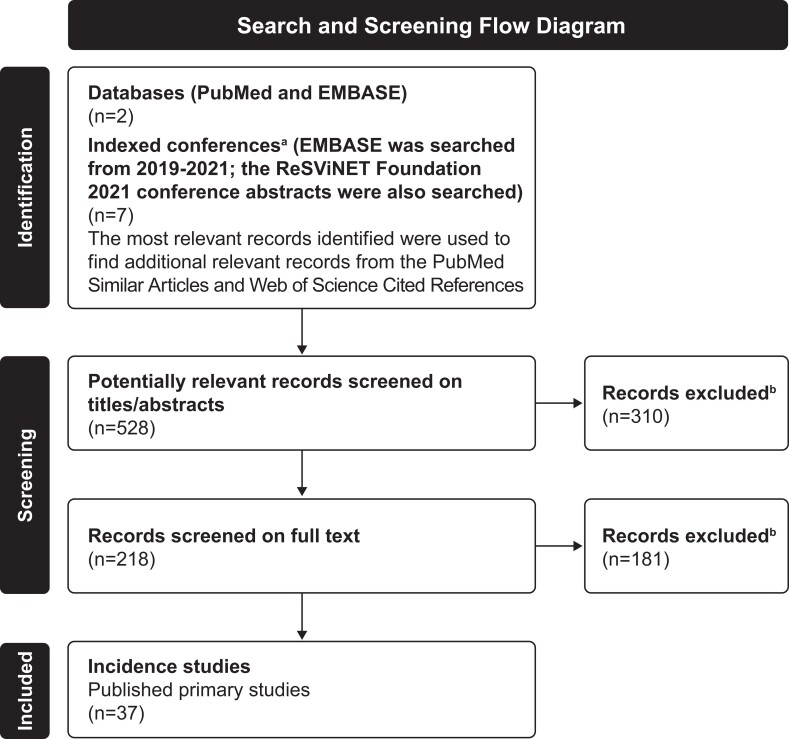
Study search and screening flow diagram. ^a^Indexed conferences included the following: American Thoracic Society (2019–2021 abstracts indexed); CHEST Annual Meeting (2019–2021 abstracts indexed); Infectious Diseases Society of America (IDWeek; 2019–2021 abstracts indexed); the Professional Society for Health Economics and Outcomes Research (ISPOR; 2019–2021 abstracts are indexed); CHEST Congress (2019–2020 abstracts indexed); and European Respiratory Society (2019–2021 abstracts indexed). ^b^Exclusion criteria for records were lack of data on respiratory syncytial virus in the study; no estimate of incidence available; no adults in the study sample; and the record was a case report, commentary, editorial, published systematic literature review, frequency study, or unpublished primary study. Diagram adapted from Page et al [58].

All potentially relevant records were screened, and incidence data were extracted by age group where available. Age limits were used to restrict the search to adults. Observational studies reporting the cumulative incidence or incidence rate of RSV were included; commentaries, cross-sectional studies, case series, case reports, studies not reporting data on adults, or studies reporting only the proportion of RSV-positive cases were excluded (Figure 1).

## RESULTS

### Search Results and Study Characteristics

**Figure 2.**
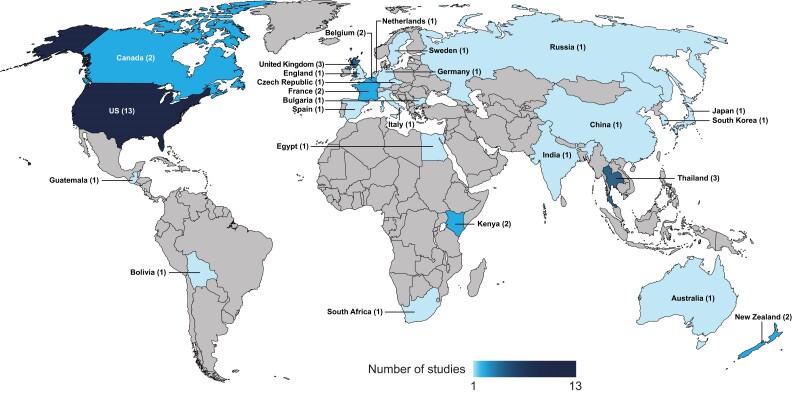
Geographic distribution of respiratory syncytial virus (RSV) incidence studies. The number of studies in each geographic region is indicated in parentheses. Estimates of RSV incidence in community-based populations: Belgium (n = 1), the Netherlands (n = 1), United Kingdom (n = 1), India (n = 1), and Thailand (n = 1). Estimates of RSV incidence in medically attended populations: United States (n = 9), Canada (n = 1), Bolivia (n = 1), Guatemala (n = 1), Belgium (n = 1), United Kingdom (n = 1), England (n = 1), China (n = 1), Thailand (n = 2), Australia (n = 1), New Zealand (n = 1), South Africa (n = 1), Kenya (n = 2), and Egypt (n = 1). Estimates of RSV incidence in both community and medically attended populations: United States (n = 1) and Japan (n = 1). Estimates of RSV incidence in populations with underlying conditions: United States (n = 7), Canada (n = 1), France (n = 2), Spain (n = 1), United Kingdom (n = 2), Germany (n = 1), Italy (n = 1), Sweden (n = 1), Bulgaria (n = 1), Czech Republic (n = 1), Russia (n = 1), South Korea (n = 1), Thailand (n = 1), New Zealand (n = 1), and South Africa (n = 1).

**Figure 3.**
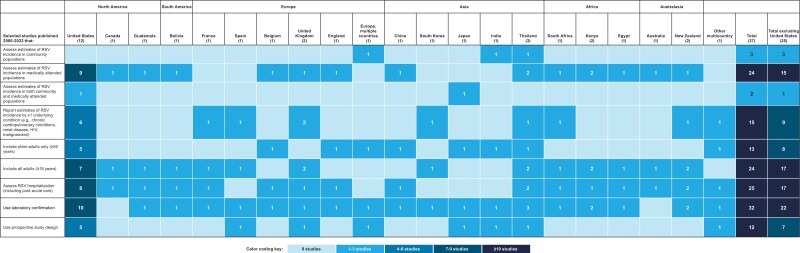
Gap analysis and observation studies reporting incidence of RSV. Numbers in parentheses represent total number of studies for the given region. Abbreviations: HIV, human immunodeficiency virus; RSV, respiratory syncytial virus.

Our search generated 528 potentially relevant articles (Figure 1). Following screening, 37 primary studies reporting RSV incidence in community populations (n = 3; Table 1) [15–17], medically attended populations (n = 24; Table 1) [8, 18–40], community and medically attended populations (n = 2; Table 1) [41, 42], and populations with underlying conditions (n = 15; 8 unique studies and 7 studies overlapping with the above categories; Table 2) [8, 17, 22, 27, 28, 30, 41, 43–50] were included (Figure 1, Figure 2, and Figure 3).

**Table 2.** RSV Incidence Rates From Studies in Populations With Underlying Conditions^a^

Citation	Region	Country	Diagnostic Method	Study Description^b^	Age, y	Incidence Estimates^c,d^
Studies in populations with cardiac and pulmonary conditions						
Praphasiri et al (2021) [17]	Asia	Thailand	PCR	2015–2017 Prospective cohort n = 313 participants with underlying CPC (n = 13 cases of RSV); participants were a subsample of a larger cohort (n = 3220)		Incidence per 100 000 PYs
					≥65	2320 (1250–3950)
Fleming et al (2015) [22]	Europe	United Kingdom	ICD-10	1995–2009 Surveillance (hospital-based) National data obtained from Public Health England, Clinical Practice Research Datalink, Hospital Episode Statistics, and the Office of National Statistics databases		Mean seasonal incidence per 100 000 population with COPD (outpatient) (range)
					18–49	23 (14–31)
					50–64	77 (49–100)
					65–74	136 (72–206)
					≥75	82 (44–121)
						Mean seasonal incidence per 100 000 population with COPD (hospitalization) (range)
					18–49	0 (0–0)
					50–64	14 (10–17)
					65–74	45 (32–52)
					≥75	75 (55–88)
						Mean seasonal incidence per 100 000 population with any underlying condition (outpatient) (range)
					18–49	1344 (924–1598)
					50–64	2221 (1581–2561)
					65–74	2508 (1764–2905)
					≥75	2860 (1948–3441)
						Mean seasonal incidence per 100 000 population with any underlying condition (hospitalization) (range)
					18–49	10 (8–13)
					50–64	102 (72–117)
					65–74	180 (127–208)
					≥75	338 (250–405)
Prasad et al (2021) [49]	Oceania	New Zealand	PCR	2012–2015 Surveillance (hospital-based) Cases of RSV: n= 227		Seasonal incidence per 100 000 population, COPD (hospitalized)
					18–49	NR
					50–64	69.9 (49.0–90.8)
					65–80	135.2 (101.8–168.6)
						Seasonal incidence per 100 000 population, asthma (hospitalized)
					18–49	13.6 (8.6–18.6)
					50–64	49.8 (36.3–63.3)
					65–80	119.6 (92.1–147.1)
						Seasonal incidence per 100 000 population, CHF (hospitalized)
					18–49	112.2 (10.5–213.9)
					50–64	79.3 (27.1–131.5)
					65–80	137.4 (81.9–192.9)
						Seasonal incidence per 100 000 population, CAD (hospitalized)
					18–49	33.4 (4.8–62.0)
					50–64	55.0 (33.5–76.6)
					65–80	72.9 (51.5–94.3)
Falsey et al (2019) [45]	Multiple	Bulgaria Canada Czech Republic France Germany Italy Russia Sweden United States	PCR or serologic test	2011–2014 Prospective cohort n = 445 study participants with COPD or CHF enrolled; n = 42 RSV-associated illnesses during the study period		Incidence per 100 000 patient-seasons (hospitalized)
					≥50	1320 (680–2300)
						Incidence per 100 000 patient-seasons (outpatient)
					≥50	3320 (2240–474 040)
						Incidence per 100 000 patient-seasons (all patients)
					≥50	4680 (3370–6320)
Falsey et al (2005) [41]	North America	United States	PCR	1999–2003 Prospective cohort n = 540 persons who had an underlying condition (COPD or CHF)		Incidence per 100 000 PYs
					≥21	18 000
Branche et al (2022) [8]	North America	United States	PCR	2017–2020 Surveillance (hospital-based) n = 10 860 who met case definition for RSV testing; n = 1039 patients with RSV; approximately half of patients in the study had an underlying cardiac or pulmonary condition		Annual incidence per 100 000 population with COPD (hospitalized)
					18–49	24.9–46.8
					50–64	204.8–210.3
					≥65	529.2–1077.4
						Annual incidence per 100 000 population with asthma (hospitalized)
					18–49	14.7–15.6
					50–64	90.2–110.9
					≥65	261.4–369.9
						Annual incidence per 100 000 population with CAD (hospitalized)
					18–49	7.8–50.7
					50–64	154.0–168.2
					≥65	517.0–554.8
						Annual incidence per 100 000 population with CHF (hospitalized)
					20–39	115.0–295.2
					40–59	231.6–485.8
					60–79	508.5–688.6
					≥80	999.9–1405.2
Griffin et al (2002) [46]	North America	United States	Culture	1995–1999 Retrospective cohort		Mean seasonal incidence per 100 000 population (hospitalized)
					50–64	1100 (710–1480)
					≥65	1770 (1160–2390)
Nolen et al (2020) [30]	North America	United States	PCR	2016–2018 Surveillance (hospital-based) n = 1163 patients with underlying cardiopulmonary conditions; n (weighted) = 10 patients with RSV		Annual incidence per 100 000 population (hospitalized)
					≥18	860 (412–1581)
Studies in populations with renal disease, diabetes, and HIV						
Prasad et al (2021) [49]	Oceania	New Zealand	PCR	2012–2015 Surveillance (hospital-based) Cases of RSV: n = 227		Seasonal incidence of RSV-associated hospitalization per 100 000 population, DM (hospitalized)
					18–49	15.2 (6.6–23.8)
					50–64	16.2 (9.6–22.8)
					65–80	52.8 (36.1–69.4)
						Seasonal incidence of RSV-associated hospitalization per 100 000 population, renal disease (hospitalized)
					18–49	NR
					50–64	87.7 (6.7–168.6)
					65–80	39.7 (15.2–94.6)
Branche et al (2022) [8]	North America	United States	PCR	2017–2020 Surveillance (hospital-based) n = 10 860 who met case definition for RSV testing; n = 1039 patients with RSV; approximately half of patients in the study had an underlying cardiac or pulmonary condition		Annual incidence per 100 000 population with DM (hospitalized)
					18–49	65.4–83.4
					50–64	113.5–116.8
					≥65	323.1–501.8
Moyes et al (2017) [28]	Africa	South Africa	PCR	2009–2012 Surveillance (hospital-based) n = 5380 participants, of which 228 had HIV		Incidence per 100 000 population, 2010
					18–44	106 (90–124)
					45–64	141 (100–193)
					≥65	390 (80–1134)
						Incidence per 100 000 population, 2011
					18–44	60 (48–74)
					45–64	137 (97–188)
					≥65	102 (25–569)
						Incidence per 100 000 population, 2012
					18–44	90 (76–106)
					45–64	198 (151–256)
					≥65	482 (156–1124)
Studies in populations with hematologic malignancies						
D’Angelo et al (2016) [44]	North America	United States	PCR	2009–2013 Retrospective cohort n = 118 patients who received SCT; n = 6 cases of RSV during follow-up		Cumulative incidence within 1 y after transplantation
					≥50	5.1%
Martino et al (2005) [48]	Europe	Spain	IF and culture	1999–2003 Prospective cohort n = 386 patients who received SCT; n = 19 cases of RSV during follow-up		Cumulative incidence within 2 years after transplantation among patients receiving allogeneic SCT
					≥18	7.0% (2.1%–12.6%)
						Cumulative incidence within 2 years after transplantation among patients receiving autologous SCT
					≥18	2.9% (0.9%–4.9%)
Hong et al (2017) [47]	Asia	South Korea	PCR	2007–2011 Retrospective chart review n = 1038 patients who received SCT; n = 31 cases of RSV during follow-up		Cumulative incidence within 100 d after transplantation
					≥18	6.9% (0.0%–16.1%)
						Cumulative incidence within 1 y after transplantation
					≥18	21.6% (6.3%–36.9%)
Chakrabarti et al (2002) [43]	Europe	United Kingdom	IF and culture	1997–2001 Prospective cohort n = 83 patients who received SCT; n = 13 cases of RSV, of which 7 cases occurred during follow-up		Cumulative incidence within 30 d after transplantation
					≥18	2.4%
						Cumulative incidence within 100 d after transplantation
					≥18	6.0%
Studies in populations with solid organ malignancies						
Testaert et al (2021) [50]	Europe	France	PCR or IF	2011–2019 Retrospective cohort n = 424 patients who received lung transplantation; n = 77 cases of RSV during follow-up		Incidence per 100 000 PYs (hospitalized or not hospitalized)
					≥18	2500 (1800–3600)
						Incidence per 100 000 PYs (hospitalized)
					≥18	2000 (1300–2900)
Studies in populations with any underlying high-risk condition						
Mesa-Frias et al (2022) [27]	North America	United States	ICD-9 and ICD-10	2000–2020 Surveillance (hospital-based) n = 36 000 patients with RSV (Optum database); n = 81 861 patients with RSV (MarketScan database)		Annual range of incidence per 100 000 population based on Optum data (outpatient)
					18–59	41.3–135.9
						Annual range of incidence per 100 000 population based on MarketScan data (outpatient)
					18–49	46.3–112.4
Fleming et al (2015) [22]	Europe	United Kingdom	ICD-10	1995–2009 Surveillance (hospital-based) National data obtained from Public Health England, Clinical Practice Research Datalink, Hospital Episode Statistics, and the Office of National Statistics databases		Mean seasonal incidence per 100 000 population with any underlying condition (hospitalization) (range)
					18–49	10 (8–13)
					50–64	102 (72–117)
					65–74	180 (127–208)
					≥75	338 (250–405)

Abbreviations: ARI, acute respiratory illness; CHF, congestive heart failure; CI, confidence interval; COPD, chronic obstructive pulmonary disease; CPC, cardiopulmonary condition; CRPD, Clinical Practice Research Datalink; CVD, cardiovascular disease; DM, diabetes mellitus; ESRD, end-stage renal disease; ICD-9, International Classification of Diseases, Ninth Revision; ICD-10, International Classification of Diseases, Tenth Revision; IF, Immunofluorescence; LRTI, lower respiratory tract infection; n, sample size; NR, not reported; PY, person-year; RSV, respiratory syncytial virus; SARI, severe acute respiratory illness; SCT, stem cell transplantation; URTI, upper respiratory tract infection.

^a^Table is ordered by region and country, alphabetically.

^b^Study period is given as year(s) over which time the study was conducted.

^c^Incidence estimates listed are incidence rates or cumulative incidence, as available in the original publications. Unless otherwise stated, incidence estimates are given per 100 000 population or per 100 000 person-years, based on available study data.

^d^Interval estimates are reported if available from the original study source documents. Interval estimates are 95% confidence intervals unless otherwise stated.

### Incidence of RSV in Community-Based Populations

Of the studies identified, 3 reported RSV incidence in community populations [15–17]. In a prospective cohort study conducted in Belgium, the Netherlands, and the United Kingdom during 2 RSV seasons, seasonal cumulative RSV incidence (polymerase chain reaction [PCR]-confirmed) in adults (≥60 years) was 2.1% (95% confidence interval [95% CI], 1.0%–3.7%) in 2017–2018 and 4.9% (95% CI, 3.2%–7.1%) in 2018–2019; of the participants recruited, approximately 67% had a comorbid condition (Table 1) [15].

Similarly, in a prospective cohort study in India (2015–2017), the PCR-confirmed RSV incidence per 100 000 person-years was 260 (95% CI, 50–810) among adults aged 60–64 years, 760 (95% CI, 240–1470) among adults 65–74 years, and 1010 (95% CI, 270–2230) among adults ≥75 years (Table 1) [16]. In another prospective cohort study conducted in Thailand (2015–2017) in community-dwelling adults (≥65 years), the estimated PCR-confirmed RSV incidence per 100 000 person-years was 1230 (95% CI, 960–1560) [17].

In summary, there are few studies on RSV incidence in community-based populations, with only 1 stratified by age group, and none in younger adults.

### Incidence of RSV in Medically Attended Populations

Of the studies estimating RSV incidence in medically attended populations (n = 24), 11 were conducted in North America (Canada, Guatemala, and the United States) [8, 24–27, 30, 34, 36, 38, 39], 1 in South America (Bolivia) [20], 4 in Africa (Kenya, Egypt, and South Africa) [18, 21, 28, 32], 3 in Europe (Belgium, England, and the United Kingdom) [22, 35, 37], 3 in Asia (China and Thailand) [19, 23, 29], and 2 in Oceania (New Zealand and Australia) [31, 33] (Table 1). Most studies (17/24) confirmed RSV using PCR; however, several studies confirmed RSV infections using immunofluorescence (IF), serological tests, culture, or International Classification of Diseases (ICD)-9/ICD-10 codes. The annual incidence of RSV-associated hospitalizations and outpatient visits (per 100 000 population) among adults ranged from 0 to 1040 and 20 to 2320 cases, respectively [20, 21, 24, 27, 28, 30, 33, 34, 36, 38, 39]. The seasonal incidence of RSV-associated hospitalizations and outpatient visits (per 100 000 population) among adults ranged from 4 to 666 and 677 to 2175, respectively [8, 22, 25, 35, 40, 49]. The incidence of RSV-associated hospitalizations and outpatient visits (per 100 000 person-years) among adults ranged from 1.9 to 130 and 0 to 517, respectively [18, 19, 23, 26, 29, 32, 37]. The annual incidence of hospitalizations (per 100 000 population) in adults aged 50–64 years and ≥65 years ranged from 0 to 170 and 0 to 1040 cases, respectively [19–23, 26, 28–33, 35–40]. The annual incidence of RSV-associated outpatient visits (per 100 000 population) in adults aged 50–64 years and ≥65 years ranged from 128 to 1450 and 37 to 2320 cases, respectively [21, 22, 24–27, 32, 39]. A few studies reported person-time incidence [23, 26, 29, 32, 37]. The incidence of hospitalizations (per 100 000 person-years) in adults aged 50–64 years and ≥65 years ranged from 7 to 40 and 5 to 130 cases, respectively. The incidence of RSV-associated outpatient visits (per 100 000 person-years) in adults aged 50–64 years and ≥65 years was 15 and 19 cases, respectively. The highest incidences of RSV-associated hospitalizations and outpatient visits were reported in older adults (≥65 years).

### Incidence of RSV in Mixed Populations (Community and Medically Attended)

Two studies were identified that reported RSV incidence in mixed populations (Table 1). A prospective cohort study in Japan (2019–2020) among adults (≥65 years; living either in the community or assisted living) reported the estimated the annual cumulative incidence of RSV-ARI (PCR-confirmed) to be 2.4% (95% CI, 1.5%–3.6%) [42]. When stratified by age group, the estimated cumulative incidences were 2.6% (95% CI, 1.4%–4.3%), 2.1% (95% CI, 0.9%–4.1%), and 2.7% (95% CI, 0.3%–9.4%) among adults aged 65–74, 75–84, and ≥85 years, respectively [42].

A prospective cohort study in the United States (1999–2003) estimated RSV incidence in healthy older adults (mean age = 75 years) and hospitalized patients (mean age = 75 years) [41]. RSV disease (confirmed by PCR or serologic assay) occurred annually in 3% to 7% of the older adult cohort; the estimated incidence rate was 10 800 per 100 000 person-years [41]. The estimated combined cumulative RSV incidence (older adult plus hospitalized cohorts) was 5.5% [41].

Additionally, a multicountry (Europe and United States) cohort study was identified in the ReSViNET Foundation conference program that reported the RSV incidence in community-dwelling adults (≥50 years) and older adults (≥65 years) living in long-term care facilities during the 2019–2020 RSV season [51]. Among community-dwelling adults, the estimated seasonal cumulative RSV incidences (confirmed by PCR or serologic assay) were 1.8% (95% CI, 1.0%–3.1%) and 1.7% (95% CI, 0.8%–3.2%) in those aged ≥50 and ≥60 years, respectively [51]. Among adults living in long-term care, the estimated cumulative incidence was 2.3% (95% CI, 0.9%–4.7%) [51].

In summary, few studies reported RSV incidence in mixed samples, with all studies focusing on adults (≥50 years).

### Incidence of RSV in Populations With Comorbid or Underlying High-Risk Conditions

#### Cardiac and Pulmonary Conditions

Studies conducted in Thailand, the United Kingdom, the United States, New Zealand, and 1 multiregion study estimated RSV incidence in populations with underlying cardiac, pulmonary, or cardiopulmonary conditions (Table 2) [8, 17, 22, 30, 41, 46, 49]. A prospective cohort study in Thailand (2015–2017) reported the estimated RSV incidence (PCR-confirmed) to be 2320 (95% CI, 1250–3950) per 100 000 person-years in adults (≥65 years) who had ≥1 underlying cardiopulmonary condition [17]. The adjusted incidence rate ratio comparing adults with cardiopulmonary conditions to those without was 2.0 (95% CI, 1.1–3.8).

A surveillance study in the United Kingdom reported that the mean seasonal incidence of RSV-associated hospitalizations per 100 000 population with COPD ranged from 0 (adults, 18–49 years) to 75 (adults, ≥ 75 years); the mean seasonal incidence of RSV-associated outpatient visits per 100 000 population with COPD ranged from 23 (adults, 18–49 years) to 136 (adults, 65–74 years) [22]. A study conducted in New Zealand (2012–2015) estimated the incidence of RSV-associated hospitalizations (PCR confirmed) among adults (≥18 years) with COPD, asthma, CHF, and coronary artery disease (CAD) [49]. The seasonal incidence of hospitalizations (per 100 000 population) varied by age group and condition, although the seasonal incidence of RSV was highest in adults aged 65–80 years for each condition. The highest overall estimated seasonal incidence was reported in adults aged 65–80 years with CHF (137.4 per 100 000 population) [49]. A global multicountry study in adults (≥50 years) with COPD or CHF reported the estimated incidences of RSV-associated hospitalizations and outpatient visits (confirmed by PCR or serologic assay) to be 1320 (95% CI, 680–2300) and 3320 (95% CI, 2240–4740) per 100 000 patient-seasons, respectively [45].

Four studies from the United States reported RSV incidence among populations with cardiopulmonary conditions. The estimate as high as 18 000 per 100 000 person-years was reported in patients with COPD or CHF in a prospective cohort study [41]. This high incidence may be due to the high proportion of older adults in the cohort (73% aged ≥65 years) and those with a history of smoking (>81%); additionally, this population had a high monthly exposure to children and was considered high care-seeking. Another surveillance study in the United States estimated the ranges of annual incidence (per 100 000 population hospitalized) for RSV-associated ARI (PCR-confirmed) among adults with comorbidities [8]. The highest incidence range (529.2–1077.4 cases) was observed among adults (≥65 years) with COPD, whereas the lowest range (14.7–15.6 cases) was observed among adults (18–49 years) with asthma; for all underlying conditions, estimates were highest for older adults (≥65 years) [8]. However, the incidence rate ratio comparing the RSV hospitalization rates among adults with versus without COPD, asthma, CHF, and CAD were generally comparable among age groups, underscoring the importance of underlying conditions across the lifespan [8]. A retrospective cohort study in Tennessee reported the estimated seasonal incidence (per 100 000 population) of RSV-associated hospitalizations (culture assay confirmed) among adults with chronic lung disease to be 1100 (95% CI, 710–1480) among those aged 50–64 years, and 1770 (95% CI, 1160–2390) among those aged ≥65 years [46]. A study in Alaska reported the estimated incidence (per 100 000 population) of RSV-associated hospitalizations (PCR-confirmed) among adults (≥18 years) with chronic lung disease to be 860 (95% CI, 412–1581) [30].

#### Renal Disease, Diabetes, and Human Immunodeficiency Virus

A surveillance study in New Zealand reported the estimated seasonal incidence (per 100 000 population) of RSV-associated hospitalizations (PCR confirmed) in patients with end-stage renal disease and diabetes mellitus (Table 2) [49] to be 87.7 (95% CI, 6.7–168.6) and 16.2 (95% CI, 9.6–22.8) among adults aged 50–64 years, respectively, and 39.7 (95% CI, 15.2–94.6) and 52.8 (95% CI, 36.1–69.4) among adults aged 65–80 years, respectively [49]. The incidence (per 100 000 population) of RSV-associated hospitalizations was 15.2 (95% CI, 6.6–23.8) in those aged 18–49 years with diabetes mellitus [49]. A US study estimated the RSV incidence per 100 000 population among adults with diabetes to be 65.4 to 83.4 (adults, 18–49 years), 113.5 to 116.8 (adults, 50–64 years), and 323.1 to 501.8 (adults, ≥ 65 years) [8].

One surveillance study conducted in South Africa (2009–2012) reported the annual incidence (per 100 000 population) of RSV-associated hospitalizations (PCR confirmed) in a population of adults with (Table 2); this incidence ranged from 60 to 106 (adults, 18–44 years), 137 to 198 (adults, 45–64 years), and 102 to 482 (adults, ≥ 65 years) [28]. Comparing individuals with to those without HIV, the age-adjusted relative risk of RSV-associated hospitalizations ranged from 12 to 18 over the study period [28].

#### Hematologic Malignancies

Four studies reported RSV incidence among adults with hematologic malignancies: 1 study in North America [44], 2 in Europe [43, 48], and 1 in Asia [47] (Table 2). A retrospective study conducted in the United States estimated RSV incidence (PCR confirmed) in patients (50–73 years) who had received stem cell transplantation (SCT) between 2009 and 2013 to be 5.1% within 1 year of transplantation [44]. A prospective cohort study in Spain (1999–2003) estimated RSV incidence (IF and culture assay confirmed) to be 7.0% (95% CI, 2.1%–12.6%) and 2.9% (95% CI, 0.9%–4.9%) among patients (19–71 years) receiving allogeneic SCT and autologous SCT, respectively [48]. Another retrospective study in patients (mean age = 44 years) receiving SCT in South Korea (2007–2011) estimated RSV incidence (PCR confirmed) to be 6.9% (95% CI, 0.0%–16.1%) within 100 days and 21.6% (95% CI, 6.3%–36.9%) within 1 year of transplantation [47]. A prospective cohort study in the United Kingdom estimated RSV incidence (IF and culture assay confirmed) in patients (18–59 years) who had undergone non-myeloablative conditioning (1997–2001) to be 2.4% and 6.0% within 30 and 100 days of transplantation, respectively [43].

In summary, participants with hematologic malignancies are at high risk of RSV disease; the cumulative incidence was 2.4% to 21.6% across identified studies, with follow-up periods ranging from 30 days to 2 years [43, 44, 47, 48].

#### Solid Organ Malignancies

One retrospective study in France (2011–2019) among adults (34–54 years) who received lung transplantation (Table 2) [50] estimated the RSV incidence (confirmed by PCR or IF) to be 2500 (95% CI, 1800–3600) per 100 000 person-years, and the of RSV-associated hospitalization incidence to be 2000 (95% CI, 1300–2900) per 100 000 person-years; notably, the latter declined with each subsequent year after transplantation [50].

#### Any Underlying High-Risk Condition

Two studies estimated the incidence of RSV disease among high-risk individuals with any underlying condition (Table 2) [22, 27]. A surveillance study in the United States among high-risk outpatients (18–59 years) with ≥1 diagnosis of asthma, COPD, CHF, CAD, HIV, or an impaired immune system reported an annual incidence of medically attended RSV (based on ICD-9/ICD-10 codes) of 41.3 to 135.9 per 100 000 population [27]. A second study in the United Kingdom (1995–2009) estimated the incidence of RSV-associated hospitalizations and outpatient visits (based on ICD-10 codes) among adults (≥18 years) with a range of underlying chronic conditions [22]. The average seasonal incidence of RSV-associated hospitalizations ranged from 3 (18–49 year olds) to 116 (adults, ≥75 years) among those without underlying conditions, and from 10 (18–49 year olds) to 338 (adults, ≥75 years) among those with underlying conditions [22]. Similarly, the average seasonal incidence of RSV-associated outpatient visits per 100 000 population increased with age and among those with underlying conditions [22].

Overall, studies indicate that RSV incidence is particularly high in adults with underlying high-risk health conditions, with some differences in incidence observed by age group. The highest RSV incidences were observed in populations with a history of transplantation and in populations with severe underlying cardiopulmonary conditions [8].

## DISCUSSION

This review identified observational studies reporting RSV incidence in adults (≥18 years) in diverse populations globally. As studies varied in the case definitions used, the type of incidence reported, the time frame of reported incidence, and the age groups used, comparing results across studies is challenging. Overall, RSV incidence increased with age in all populations, with the highest rates observed in older adults with underlying conditions. Most evidence on RSV incidence was from high-income regions, with relatively fewer data available from low/middle-income (LMI) regions (particularly the Middle East, Central Europe, Central America, South America, and most of Africa).

Case definitions for RSV disease varied across studies. The case definition of RSV-associated ARI often required the presence of fever and cough/sore throat, although some definitions included other signs/symptoms (increased sputum production, runny nose, myalgia, chills, or abnormal white blood cell count). Fever with cough/sore throat reflects a standard surveillance definition for influenza-like illness [52], which may miss RSV cases, as fever does not always occur during RSV infection. Studies utilizing this case definition may underestimate RSV incidence in adults. Furthermore, studies using ICD codes to identify RSV cases may also underestimate incidence [12].

The presentation of incidence (person-time incidence or cumulative incidence) varied across studies, making direct cross-study comparisons with different denominators challenging. However, for better comparability, data from some studies could be reanalyzed using an alternative denominator for better comparability. Additionally, incidence was either reported on an annualized or a seasonal basis. Studies in geographies where RSV typically demonstrates seasonality (eg, October-March in the United States) would generally underestimate RSV incidence if estimating incidence rate on an annualized basis; a person would accumulate person-time at risk, inflating the denominator, while not being at risk of RSV disease, which keeps the numerator constant. However, reporting the incidence proportion rather than the incidence rate on annualized basis in geographies that have distinct seasonality should have minimal impact on annual and seasonal estimates.

While most studies included >1 age group, age group categorization was highly variable between studies. Results were typically stratified by 10-year (eg, 65–74) or 15-year (eg, 50–64) age bands; in other instances, 20-year, 25-year, or open bands (eg, ≥ 18 years) were used, especially in studies of older adults. In community populations and medically attended populations, RSV incidence increased with age, with the highest incidence reported among older adults [8, 16, 20, 22–30, 33–36, 39, 40, 46, 49]. Adults with comorbid/underlying conditions are at increased risk of severe RSV disease, which can lead to hospitalization and death [2, 3]. Among persons with underlying conditions, RSV incidence typically increased with age [8, 22, 28, 46, 49]; however, 1 study found a higher incidence in younger adults (20–39 years) with CHF compared with older adults with CHF [8]. Overall, among all included studies, older adults (especially those aged ≥80 years) with an underlying condition had the highest incidence of RSV. Further investigation is needed to clarify whether older adults are indeed more likely to experience RSV compared with their younger counterparts, or if this finding is instead due to a greater likelihood of RSV detection due to the aging-associated increases in medically attended symptomatic disease and underlying medical conditions [38].

Because routine RSV testing is infrequently performed, it may be difficult to obtain accurate incidence estimates for adults in high- or low-income regions due to underdetection or underreporting. The accuracy of incidence estimates may also be affected by methodologic challenges (eg, differences in case ascertainment approaches and limitations in laboratory testing) as well as structural (eg, access to care) or behavioral factors (eg, care-seeking behaviors). Case ascertainment may be affected by inappropriate case definitions and inconsistent/sporadic RSV testing. A global systematic review found higher RSV-associated hospitalization rates among older adults from high-income compared with LMI regions, which could be explained by differences in care-seeking behaviors or access to care [1]. A surveillance study in Kenya showed that the incidence of outpatient visits for RSV disease was higher after adjusting for care-seeking behavior [21]. Research from LMI regions suggests that care-seeking behaviors may be more related to symptom severity than proximity to healthcare facilities [32].

Most evidence on RSV incidence is from high-income regions, including Canada, the United States, Japan, Australia, New Zealand, and a few countries in Western Europe. There are few incidence data overall from most LMI regions, where evidence is concentrated in countries in southeast Asia and northeast/eastern Africa.

The reported RSV incidence data should be considered in light of the potential impact of tests used to detect the virus and surveillance tools used to identify infected patients. PCR testing of nasopharyngeal swabs is the most sensitive and commonly used method in hospitals, and was the diagnostic method in 22 of the studies captured in this analysis. However, this method is known to be less sensitive for upper than lower respiratory tract infections, largely due to inadequate viral load on nasopharyngeal specimens (particularly in adults), resulting in missed cases and underdiagnosis [53, 54]. Addition of multiple specimen types (eg, saliva, sputum, serum) was found to increase the RSV detection rate, while testing of all 4 specimen types doubled the diagnostic yield [53, 54]. Improved methods of detecting RSV may provide a more accurate account of incidence rates and reveal the true extent of the disease burden.

Note should also be taken of reports in which ICD-10 coding alone was used for RSV surveillance [22, 27, 33], because this has been demonstrated to underestimate the disease burden [55, 56]. A study in Germany [55] demonstrated that while the use of RSV-specific ICD-10 codes for disease surveillance was appropriate for identifying age groups at high risk of RSV and for monitoring trends and RSV seasonality, their use led to the underestimation of the actual number of (laboratory-confirmed) RSV infections in the primary-care context. This issue was mitigated by combining RSV-specific ICD-10 codes with general ICD-10 codes for acute lower respiratory tract infection [55].

In conclusion, observational studies in adult populations showed that RSV incidence increases with advancing age and among those with underlying high-risk conditions. Several gaps in the literature were identified, including the need for more evidence on RSV incidence in community-based populations and LMI regions. Furthermore, consistency across studies in the common surveillance case definition used for RSV testing, the case definition of RSV-associated ARI, and the measurement and presentation of RSV incidence would facilitate comparing the incidence estimates across time and geographies (Figure 2). Such evidence is critical to understand the potential impact of RSV vaccines in adult populations globally [57].

As adult RSV vaccines are introduced, it is increasingly important to understand the baseline burden of RSV disease across a variety of geographies and population characteristics so that their impact can be accurately estimated as deemed relevant to the populations who will receive them. For future studies seeking to measure the incidence of RSV disease in adult populations, accurate case ascertainment is critical, and should be accompanied by clear descriptions of the measurements (eg, incidence rate or incidence proportion), time periods (eg, annual or seasonal, and over what calendar period), and characteristics of the underlying populations involved.

## Notes

***Acknowledgments.*** Louise Falzon, BA, PGDipInf, a consultant of Bohn Epidemiology, LLC, developed the search strategy and conducted the literature search. Medical writing and editorial assistance were provided by Jared Mackenzie, PhD, and Louansha Nandlal, PhD, of MEDiSTRAVA, in accordance with Good Publication Practice (GPP3) guidelines, funded by Moderna, Inc, and under the direction of the authors.

***Author contributions.*** All authors were involved in data collection and analyzed or interpreted the data. All authors provided writing, review, or intellectual contributions, and approved the final draft.

***Financial support*.** This work was supported by Moderna, Inc.
